# The Auto-Protective Resources of the Body—A New Foundation for Scientific Therapeutics*Read by invitation before the American Therapeutic Society and the Philadelphia Branch of the American Pharmaceutical Association, May 7, 1908.

**Published:** 1908-10

**Authors:** Charles E. De M. Sajous

**Affiliations:** Philadelphia


### TEXAS MEDICAL JOURNAL.

**Established July, 1885.**

F. E. DANIEL, M. D., - - - - Editor, Publisher and Proprietor

PUBLISHED MONTHLY.—SUBSCRIPTION $1.00 A YEAR.

VOL. XXIV. AUSTIN, OCTOBER, 1908. No. 4.

The publisher is not responsible for the views of contributors.

### Selected.

### The Auto-Protective Resources of the Body—A New Foundation for Scientific Therapeutics.*

BY CHARLES E. DE M. SAJOUS, M. D., PHILADELPHIA.

[From Monthly Cyclopedia of Practical Medicine, May, 1908.]

Prof. John H. Musser, in a paper published three years ago in the *American Journal of Pharmacy*, stated that "just as the present compares with twenty years ago, one can see less and less of the use of drugs." While predicating from this deduction that twenty years hence "a minimum of drugs" would be used, he cited broncho-pneumonia and tuberculosis as examples of the value of rest, fresh air, and proper food. Sir Frederick Treves more recently gave vent to similar views; while recognizing that "the habit of taking medicines lies deep down in the hearts of the people," he too thought that the use of drugs would "be replaced by simple living, suitable diet, plenty of sun, and plenty of fresh air." Similar conclusions are often published by men of high standing. And yet can they, in truth, dispense with drugs? No experienced practitioner will deny that nine-tenths of our professional usefulness is based on pharmaceutical remedies. Not only do our patients crave active, militant protection and relief in the hour of suffering, but the physician knows through the teachings of practical experience that drugs are his legitimate and often trustworthy weapons of war-

*Read by invitation before the American Therapeutic Society and the Philadelphia Branch of the American Pharmaceutical Association, May 7, 1908.

fare, the strongest shield he has to interpose between his patients and the fell destroyer.

Closely allied to the air, food, and water apostles are the therapeutic nihilists who, like Professor Osler, believe in the doctrine of self-limited diseases and look on while Nature and the disease have it out. Dr. Abraham Jacobi who, in accord with the great bulk of the profession, considers expectancy as a synonym for loss of time and opportunity, characterizes this nihilistic attitude as a compound of ignorance and indolence, and as "a sin of omission which frequently rises to the dignity of a crime." Emphasizing, moreover, the truth of Dixon's doctrine that the tendency of all diseases is toward death, he urges—and sustains by illustrative cases—that lack of timely treatment is accountable for much loss of life. Indeed, we blame, and our courts sometimes punish mystic therapeutists, Christian scientists, etc., for similar reasons, and it becomes a question of whether their attempt to afford some help does not exonerate them in the presence of our own therapeutic pessimists who supply nothing—except, perhaps, a correct death certificate if the patient happens to die.

Another most regrettable feature of present-day medicine is that public confidence in our professional efficiency is on the wane, as the frequent attacks in the lay press well attest. Disregarding totally the many sides of our professional work which should command appreciation, the incalculable benefits that thousands of noble and self-sacrificing men have bestowed upon humanity, many people who, in the moment of danger, at once seek our aid are not loath to lapidate us when the occasion offers. Other countries, even the great European centers of learning, are equally militant. Thus, Professor Dieulafoy, of Paris, declared only a few months ago, that medical men are criticised on all sides: in public, in the drawingroom, at dinner parties, on the stage, in the newspapers, etc., and that books, even, are written expressly to condemn the medical profession! The normal results of this campaign are that a multitude of innocent people are increasingly driven into the hands of quacks; that systems of practice based on mysticism and misrepresentation are steadily gaining ground, and that patent medicine vendors are accumulating untold wealth at the expense of the unwary.

What is the underlying cause of all this? An analysis of the causes of defection in our own ranks is, I believe, first in order. Indeed, as stated by Llewellys F. Barker in 1900, at the close of a century during which all sciences other than medicine had advanced at an amazing pace: "With many, pharmacotherapy, as a whole, is almost moribund."

Shall we, however, with Dr. Jacobi and other distinguished men of our profession, ascribe to ignorance the loss of confidence in remedies that some of its members betray? What physician would presume to assert that mercury in syphilis, quinin in malaria, iron in simple anemia, arsenic in pernicious anemia, thyroid extract in cretinism and myxodema, antitoxin in diphtheria, digitalis in certain cardiac disorders, sodium salicylate in muscular rheumatism, strychnin in adynemia, and many other remedies I could cite, are useless? And yet, Dr. Frank Billings wrote, in 1903, that "drugs, with the exception of quinin in malaria and mercury in syphilis, are valueless as cures"; in 1907 these last vestiges of our glory must have vanished, for the president of a prominent British society, Dr. A. H. Brampton, took occasion to say, as a prelude to a personal expression of dissent: "Skepticism is in the air. Even in this society, if any daring member has introduced a subject bearing on medical treatment, it has been with an apologetic air and humble mien, well knowing that if his remarks had any reference to the utility of drugs in the treatment of disease, they would be subject to good-humored banter, and received by those sitting in the seat of the scornful with amused incredulity." May we not be dealing with an unwarranted prejudice against drugs rather than with ignorance? Again is it not among the best informed men of our professon that drug-nihilism prevails pre-eminently?

It should be attributed, it seems to me, to an entirely different cause—one, indeed, which is increasingly making itself felt from day to day. Bichat said over a century ago, "pharmacology, in its present state, is not a science fit for a methodic mind." Notwithstanding the enormous painstaking labor that has been devoted to the multitude of problems it offers, Bichat's estimate is still applicable—a fact emphasized by the oft repeated admission that therapeutics has not raised itself above the level of empiricism.

We all know, for example, that mercury is curative in syphilis; but how is this accomplished? This, as Manquat asserts in the last edition of his *Therapeutics,* "is impossible to say." Another mainstay in many diseases is iodine, not only the haloid proper, but its salts. Hare writes: "The physiological action of iodine, so far as its alterative powers are concerned, is absolutely unknown." The brightest star of late-date therapeutics, antitoxin, stands not a whit higher. H. C. Wood, after reviewing the more familiar theories of its action, including Ehrlich's theory, says:

"It must be confessed that we have no positive knowledge of the manner in which this substance acts in infectious diseases." Another truly wonderful agent—particularly in myxedema and cretinism—is thyroid extract, but, as to its mode of action, Laulanié tells us that "for the time being there is not even a clue to the solution of this problem." As to digitalis, H. C. Wood, Sr. and Jr., write: "In our experiments upon the exposed mammalian heart, we have seen in the final acts of digitalis drama happenings so curious that at present no proposed theory as to the action of the drug is sufficient." This obscurity applies more or less to all drugs administered internally.

On the whole, pharmacology, which, as shown by Bichat's contemptuous remark, was in a deplorable condtion early last century, was still, at its close, notwithstanding an enormous aggregate of sound scientific facts contributed by a host of brilliant scientists, "in a backward and unsatisfactory condition," as a great clinician, Sir Andrew Clark, once expressed it. Indeed, no one can deny that it has failed to keep pace with the other branches of medicine: Diagnosis, physiological chemistry, histology, etc., which have steadily gained for themselves a place among the medical sciences. Although the most important of all, and the one branch through which the public, the whole world, in fact gauges our actual professional efficiency, therapeutics has ever remained a mere art. A remedy is still given because it has been found more or less efficacious in this or that condition by others, and the subdivisions headed "treatment" or "therapeutics" in our text-books are mere catalogues of drugs which are stated to be "particularly useful," "most efficient," "very valuable," "commoly employed," "of great value," etc., in this or that disease, and in which not an inkling is afforded as to *how* the remedy antagonizes the morbid process. Not only does this entail a source of error at every step, since it often happens that a drug indicated in one stage of the disease may be contra-indicated in another, but it leaves the conscientious physician in constant anxiety lest the agent prescribed empirically prove more harmful than beneficial. Hence the frequent, sometimes daily change of remedies—to which shelves covered with pill boxes and small bottles attest—and, I may add, the ill-repute into which therapeutics has fallen.

Need we wonder that highly trained men, who have developed great keenness as diagnosticians and the highest attainable proficiency in pathological histology and chemistry, have lost faith in pharmacotherapy? Are we justified in attributing to ignorance their loss of faith in a system of administering remedies which is as irrational and archaic as the other departments of medicine are precise and modern? Indeed, it is merely *because pharmacotherapy has not kept pace with the immense strides of all other branches that it is, with many physicians,* as stated by Prof. Lewellys F. Barker, *"almost moribund."*

Next in order is the cause of this deplorable condition of therapeutics. Must we attribute it to the host of investigators in pharmacodynamics to whom I have referred? Even though experimental errors to which I will allude presently, might have delayed progress, I firmly believe that if there had not been insuperable obstacles in another direction, therapeutics would stand today among the most advanced medical sciences as a result of their labors. One of their number, the foremost sponsor of experimental therapeutics of our day, Horatio C. Wood, Sr., of our own city, and several equally eminent foreign colleagues, would long ago have given us a sound foundation for the intelligent use of drugs had their deductive reasoning not been handicapped at every step by a feature of the question to which I called attention last year: *The shortcomings of physiology.*

To interpret intelligently the physiological action of alteratives and tonics, which are in daily use by physicians, for example, an accurate knowledge of general metabolism, the foundation of nutrition, is necessary. What is known on this subject, according to the late Sir Michael Foster, "consists mostly of guesses and gaps." The action of cardiac stimulants includes as main phenomenon, showing of the heart through the mechanism of inhibition. Physiologists, according to Langley, "are still far from any real knowledge of the processes involved in inhibition." Diaphoretics and sialagogues activate the sweat and salivary glands by causing vaso-dilation in these organs. As Chapman says: "Though numerous explanations have been offered of the manner in which the vaso-dilator nerves act, it must be admitted that none of them are satisfactory." The study of hypnotics and anesthetics presupposes a knowledge of the process through which sleep is produced. According to Bradbury, "the phenomenon is still enveloped in mystery." Analgesics and nerve sedatives and stimulants owe their action to modifications in the character of the impulses awakened in the nerves; but as Stewart says, "What the nerve-impulse actually consists in we do not know." This factor is intimately linked, in the action of antispasmodics and convulsivants, with muscular oxidation, but Laulanié writes "we are absolutely ignorant of the mechanism of organic oxidation." Purgatives, whether their effects be due to an increase of secretory or peristaltic activity evoke them through secretory or motor nerve-paths; but, as Langley states, the relation of the enteric nervous system to the cranial nerves "is at present a matter of guesswork." Emetics suggest the need of adequate knowledge of the process of vomiting, but, according to Foster, "The nervous mechanism of vomiting is complicated and in many respects obscure." To explain the action of diuretics the mechanism through which the secretion of urine is governed should be known; "As yet," writes Landois, "only the influence of the vasomotor nerves upon the filtration of the urine from the renal vessels is known"—which means that that of the secretory nerves is not.

In brief, every important subdivision of therapeutics thus finds itself deprived of the essential physiological knowledge upon which a rational explanation of its physiological action could be poised. And this does not apply to therapeutics only; pathology is suffering, and has suffered, all along from the same cause, since, after all, the morbid effects of bacterial toxins or endo-toxins, poisons, toxic wastes, etc., act in the body as would poisonous doses of drugs persistently administered.

Is there any indication that physiologists will furnish the data we need to place pharmacology on the sound basis we so earnestly crave? I must frankly express the belief that, judging from present indications, the prospects are very discouraging. In the first place, a cursory glance through physiological literature shows that they devote very little work to the study of the physiological problems I have enumerated. In the second place, they overlook the one source of information through which their own labors could be made promptly to bear fruit, that covered by the assertions of a great Russian physiologist, Professor Pawlow, that in many instances "the physician gives a more correct verdict concerning physiological processes than the physiologist himself," and that "clinical observation will consequently always remain a rich mine of physiological facts." In the third place, I greatly regret to say that they are steadily deserting us. A physiologist, Dr. Meltzer, wrote, a few years ago: "Physiology is of medical parentage, was reared by medical men, and is still housed and fed by medical faculties. Still it is medicine against which its frequent declaration of independence is directed. Medicine is a practical science, and is too inexact, and physiology wishes to be a pure, exact science. It, therefore, tries to keep aloof from medicine, and manifests a longing for association with, or, better still, for a reduction to physics and chemistry." Time has only served to sustain this statement and to emphasize my own conviction that medicine, and particularly pharmacology, will not be redeemed from its present deplorable position as long as it will depend entirely upon physiologists for the elucidation of function.

This is not intended to mean that pharmacologists are not also open to criticism. But their shortcomings can not be ascribed to a lack of effort on their part to elucidate the multitude of problems they are expected to solve; they are due, in my opinion, to errors of procedure and of interpretation in the course of their experimental work.

First among these is the belief that drugs given by the mouth, injected hypodermically or rectally, travel to the tissues and act upon them. Text-books tell us, for instance, that digitalis excites directly the muscular fibers of the heart, and that the rise of blood-pressure and increase of pulse-volume are mainly due to the greater propulsive power with which the drug endows this organ. This is based on experiments in which the heart, partially or entirely detached from the animal, is caused to resume its beats by dropping upon it a solution of digitalin. That this experiment is misleading is suggested by various facts. The weakest solution that will act directly on the heart thus exposed is 1 part in 50,000. Now, a large therapeutic dose of digitalin by the mouth is ¼ grain, which, dissolved in the blood-mass, would make a solution four times weaker, 1 to 200,000. Hypodermically, 1-16 grain of digitalin produces distinct physiological effects, and yet this is only a solution of 1 to 800,000 in the blood-mass—a solution sixteen times weaker than is absolutely necessary to influence the exposed heart experimentally. The prevailing belief in this connection becomes absurd when to these figures we add the facts that the drug is chemically modified in the blood—when given orally, hypodermically, or rectally—long before it can reach the heart-muscle at all, and that a large proportion of the blood is constantly passing out into the lymph spaces and vessels, thus necessarily carrying a correspondingly great proportion of the drug with it. Again, why is it, if digitalis acts directly on the heart, that it affects much more actively the right side of this organ than the left? If the blood carried it to the heart-muscle, both sides should be influenced similarly. Another suggestive fact is that division of the spinal cord prevents the effects of digitalis given internally; obviously if the drug acted directly on the heart-muscle, this procedure would not prevent its action. Need we wonder that H. C. Wood, Sr. and Jr., can still say, in the last edition of their *Therapeutics,* that "at present no proposed theory as to the action of the drug is sufficient"?

There is ample experimental and clinical evidence to show that it is upon the general nerve centers that the vast majority of our drugs act, and that the phenomena witnessed are those awakened by these centers or resulting from diminution or arrest of their functional activity. As stated by Prof. Charles Richet, of Paris, "all toxics (with rare exceptions) are hardly poisonous otherwise than through their action upon the *nerve-cell.* In the organism," adds this distinguished physiologist, "the nerve-cell, to the detriment of other cells, muscular, glandular, epithelial, is the most sensitive to toxic action." Such being the case, the most sensitive nerve-cells, those of nerve centers, are the first to feel the influence of the small doses of toxic that our remedies represent, and it is because of this that such a minute dose of aconitine as 1-400 grain, for example, can depress the whole circulation, and cause muscular torpor and weakness and also, perhaps, tingling in the extremities.

A second experimental error which proves misleading in most instances is the use of anesthetics in animals in which a remedy is tried. Ergot, for instance, has been regarded as a vaso-constrictor since its action had been at all seriously studied, when certain investigators, basing their assertion on hundreds of experiments, declared that this agent did not cause vascular contraction. But an examination of their work revealed that they used ether as anesthetic, an agent which, as is well known, is a powerful vaso-constrictor. How could they possibly cause contraction of the vessels with ergot when these blood channels were already contracted to their utmost limit by the ether?

A third source of error, one which prevents progress in our knowledge of the action of drugs that affect the cardio-vascular system, is the neglect of waste products as factors in the process. Although veratrum viride, for instance, has long been considered a powerful vaso-dilator, capable of causing the blood to accumulate in the deep and large vessels, thus depleting congested organs, recent experiments have suggested that this drug in reality constricts the arteries. But analysis of these experiments shows that the older view is the correct one, and that the vaso-constriction witnessed by the experimenters was not due to the drug. The large dose used had produced sc intense a vascular dilation that catabolism of waste products in the peripheral capillaries had been inhibited; and these wastes being, as is now well known, powerful vaso-constrictors, they overcame the vaso-dilator action of the veratrum viride and produced vaso-constriction.

A fourth and very misleading error is the experimental use of excessive doses in animals to determine the action of a remedy. In a recently reported study of the therapeutic action of potassium iodide, for example, the investigator administered to his animals doses which in man would represent 346 grains! The effects produced by such doses does not only fail to exemplify the therapeutic action of the drug; they are those of intoxication. As stated by Hale White, "When a drug in moderate doses excites a function, in large doses it often paralyzes it." This applies to a large proportion of the experiments on record, and it is to this fact that the present ignorance of their physiological action must, in great part, be ascribed.

A fifth cause of confusion is the belief that because certain drugs, atropine, for example, paralyze nerve endings by acting directly upon them when locally applied, it produces the same effect through a similar process when given hypodermically or orally. If the smallest dose that will evoke a physiological effect when given internally is used to prepare a solution with the entire blood and lymph (in which the drug also circulates) of a control animal of equal weight, it will be found inert; first, because the solution obtained is entirely too weak, and, second, because it is chemically altered by the blood's antitoxic constituents. In the living animal, and particularly in the carnivora, this latter process is far more active than in shed blood, to such a degree, in fact, that blood drawn from an animal treated to a dose capable of producing marked physiological effects will prove inactive. The physiologists are the principal victims of this class of error—which applies to other alkaloids, pilocarpine, muscarine, nicotine, etc., employed by them, and many conclusions regarded by these scientists as sound are in reality defective and, therefore, misleading.

The sixth and last source of error I will submit is one which doubtless has cost many lives, namely: The assumption that certain phenomena produced by drugs are manifestations of a normal function. Digitalis, for example, is said to slow the heart by stimulating the vagus; that is to say, the mechanism of inhibition—a supposedly physiological function. But there is no ground for this assumption. In the first place, as Porter says, "the nature of the terminal apparatus by which the vagus inhibits the heart is unknown." In the second place, the coronaries and other arteries of the heart, contrary to what physiologists teach, are supplied with vasomotor nerves, as shown by microphotographs of these nerves in my work on the "Internal Secretions." Now, ample evidence is available to show that all the drugs which slow the heart do so partly by causing constriction of these cardiac arteries; that is to say, by reducing the volume of blood supplied to the heart wall. We are not witnessing a normal phenomenon, therefore, when under the influence of large doses of various cardiants the heart is slowed, but a morbid effect, weakening of the heart muscle, a factor to which the increased resistance of the general arterial system to the heart's contractions adds a cause of cardiac arrest.

Other examples could be submitted, but these will suffice, with the shortcomings of physiology previously enumerated, to suggest why therapeutics has failed to keep pace with the other branches of medical science; why, in other words, as Professor Sollman, a medical member of the Council of Pharmacy of the American Medical Association, could write, regretfully doubtless, only last month: "A generation ago therapeutics was an art, promising to develop into a science. At present it can not be classed as an art, nor as a science; it can only be classed as a confusion."

Need I further urge that new and more fruitful lines of thought are imperatively needed if the true worth of our profession, its humane purpose, and its very existence are to be perpetuated?

I believe that it is fully within our power as pharmacologists to remedy the prevailing state of things, and to raise therapeutics to the dignity of a science. At no time in the history of the world has medicine been richer in solidly grounded experimental and clinical facts, precisely those we need to attain this most ambitious of our aims. We hear much of empiricism, the stigma of medicine as an art, but, as Huxley once said: "All true science begins with empiricism, though all true science is such exactly in so far as it strives to pass out of the empirical stage." With the means now at our disposal we need only to strive, to eliminate forever from our escutcheon the blemish that "empiricism" implies. Hosts of workers, those to whom Oliver Wendell Holmes referred when he wrote,

> "Blest is he who knows no meaner strife
> Than Art's long struggle with the foes of life,"

have lived a life of devotion to suffering mankind. They may not have left us as heritage a therapeutic structure, or even the foundation for one, but they have furnished the hewn stones out of which it can be erected. It is for us to build. We may speak of confusion today; but confusion likewise prevails before each pillar, each stone, of any great edifice has found the place assigned to it by the co-ordinating mind of the architect. *Therapeutics needs architects and builders today far more than it does experimenters;* and when that fact will be thoroughly apprehended a new era for medicine as a whole will begin; one that will ultimately lead to the highest of human accomplishments: The mastery of disease in all its forms.

It is the result of an effort in this direction, begun some twenty years ago, that I am about to outline. Therapeutics may not exist as a science, but a close scrutiny of all the branches of knowledge related to medicine convinced me that with the many sources of error, physiological and pharmacological, I have submitted, once overcome, the aggregate of sound scientific data at our disposal in literature would supply the elements necessary for an accurate knowledge of the action of drugs. Physiology, cytology, embryology, and the more comprehensive branches of which they form part, zoology and botany, physiological chemistry, and other branches had to be ransacked for what elucidative facts they might contain. I was overwhelmed by the treasures these auxiliary departments of science contained. Clinical medicine especially proved itself, in keeping with the previously-quoted opinion of Professor Pawlow, "a rich mine of physiological facts."

In submitting to you the briefest possible outline of the newer conception of the action of drugs my labors have suggested, I will introduce only what evidence is absolutely necessary to make my meaning clear, referring you for the rest to the two volumes of my work on the "Internal Secretions." During the balance of the hour allotted to this paper, I will lay special stress, however, upon a conclusion which appears of capital importance: That *we should look to the auto-protective resources of the body, and the laws through which drugs influence them, for a scientific therapeutics.* And let me add that I am no longer alone in advocating this principle. In 1903, only a few months after the first volume of my work had appeared, and before he could have learned its contents, an eminent clinician, Professor Hayem, of Paris, for instance, taught that the tendency of modern practice was to return to the views of Hippocrates, who believed in the *vis medicatrix naturæ;* "therapeutic weapons are wanted," declared the French clinician, "which will reinforce the defensive power of the organism." Need I add that the entire trend of medical thought is in this direction; that the study of immunity has captivated the best minds of modern times: Pasteur, Metchnikoff, Pfeiffer, Bordet, Behring, Roux, Ehrlich, and others? I am not departing from the legitimate field of science or of sound judgment, therefore, when I urge that when a disease is due to the presence of pathogenic bacteria, their toxin, toxic wastes, or any other poison, our aim should be to so enhance the defensive properties of the blood with our remedies, that the disease-breeding agents will promptly be destroyed and converted into eliminable end-products.

Granting that the remedies capable of doing this are known, we remain in the realm of empiricism unless we can ascertain how they act; *how,* in other words, the blood is caused by certain drugs to attack and destroy both germs and poisons. This small word "how" is a far reaching one, for it is upon our ability to meet its mandates that the standard of therapeutics as a science depends.

How, then, does any agent enhance the defensive powers of the body? Are we supplied with a mechanism which enables us to ward off disease—a mechanism whose activity we can enhance at will? This is the dominant problem that my labors have aimed to solve; they showed that such a mechanism does exist, and that it is built of a set of small organs regarded by Brown-Séquard as the producers of internal secretions, but the functions of which had remained obscure, namely, the pituitary body, the thyroid gland (including the parathyroids), and the adrenals which jointly form what I have termed the "adrenal system."

A brief review of the rôle of each of these organs is necessary before the action of drugs upon them can be defined.

The *pituitary body* is one of those unfortunate structures which histologists and physiologists relegate to the waste basket as "vestigial organs" when they can not explain its functions. It is fortunate, in fact, that it is located below the brain, beyond the reach of surgeons, for there would have been a holocaust of pituitary bodies just as there has been a holocaust of appendices, ovaries, etc., unless resort to surgery had been checked by the appalling results of such a procedure. Indeed, when this organ is completely removed in adult animals, formidable symptoms ensue, the temperature and the blood pressure recede; nutrition is inhibited, as shown by rapid emaciation, the intense weakness and the lowered metabolism. Dyspnea, muscular co-ordination, interpreted by convulsions, follow, and, usually on the third day, the animal lapses into coma and dies. Conversely we find the pituitary causing opposite phenomena when it is the seat of hyperemia, hypertrophy, or tumors which render it overactive. In the early stage of acromegaly, for instance, the general nutrition and muscular power are greatly increased. The overnutrition is such, in young subjects, that their stature often reaches that of giants.

The importance of the pituitary to life is further emphasized by the fact that the morbid symptoms and death caused by its removal do not follow removal of the brain. As shown by the Cornell frog, Goltz's dog, and other examples, all the functions other than intelligence, are, after recovery, as perfectly performed as if the cerebral hemispheres were still present.

How does the pituitary so prominently influence the temperature, the blood pressure, metabolism, and nutrition, all functions which affect the entire organism?

Is it through the intermediary of a secretion as generally believed? Not a single proof is available in literature to show that the pituitary body secretes anything. There is ample evidence, however, contributed by such men as Cajal, Andriezen, Gentès, and others, to show that the pituitary is connected by nerves with the base of the brain. My researches showed, moreover, that these nerves were the beginning of a nerve path, which, passing by way of the bulb, the spinal cord, the upper sympathetic ganglia, and the splanchnic terminated in the adrenals. Not only did excitation or division of this path at intervals produce the identical phenomena observed after removal, excitation, or disease of the pituitary body, but the same procedures applied to the adrenals also provoked the same phenomena. Briefly, I found that the pituitary body and the adrenals were united by nerves, and that it was through the adrenals that the pituitary produced the various phenomena credited to it.

It becomes a question now as to how the *adrenals* awaken these remarkable effects.

Physiologists had been unable to discover the identity or source of an "internal secretion" shown by Bohr and other physiologists to be necessary to explain pulmonary respiration, in so far as the taking up of oxygen from the air was concerned. They had admittedly also failed to find the origin of 96 per cent of the substance which distributes oxygen to all the tissues, the hemoglobin. My investigations showed that it was the secretion of the adrenals which fulfilled both these functions. Being secreted into veins which open into the inferior vena cava, it inevitably reached the lungs; being a powerful reducing agent, it necessarily absorbed oxygen on being exposed to the air of the air cells. Moreover, the previously unidentified albuminous component of hemoglobin gave all the reactions of the adrenal secretion. A striking confirmation of the latter fact was recently contributed by Professor Mulon, of Paris, who found that the red corpuscles gave all the reactions of adrenalin.

The manner in which the adrenals awaken the various phenomena previously enumerated may now be accounted for. As their secretion is the substance which supplies oxygen to all tissues, their removal causes a lowering of the temperature and of the blood pressure, arrest of nutrition, emaciation, great muscular weakness, and death—precisely the symptoms that follow removal of the pituitary body, the seat of their center. Conversely, it explains how adrenal extract or adrenalin raise the temperature and the blood-pressure, *i. e.,* by enhancing oxygenation and metabolism in all tissues. The remarkable influence of the adrenals on life—in keeping with that of the pituitary body—is not only shown by the fatal effects of their removal, but also by the direct action of their products in sustaining life. As is well known, Crile, by means of injections of adrenalin in saline solution, was able to resuscitate animals fifteen minutes after all signs of life had ceased, and to keep a decapitated dog alive ten hours.

On the whole, the pituitary body through its connection with the adrenals governs the pulmonary and tissue respiration; that is to say, the life process itself.

The pituitary body regulates the functions of another set of organs, the thyroid gland and its glandules, the parathyroids. The nerves from the pituitary to these organs were discovered by de Cyon, a Swiss physiologist, who pointed out that they caused dilation of the thyroidal vessels, increasing, thereby, the functional activity of these organs.

The *thyroid and parathyroids,* in the light of my researches, play an important rôle closely related to that of the adrenals. The purpose of their secretions, acting jointly, is to increase the vulnerability or sensitiveness of all tissues, waste-products, bacteria, etc., to oxidation, by enhancing directly the inflammability of their phosphorus. In other words, it causes all these bodies to burn faster under the action of the oxygen-laden adrenal product, the albuminous hemoglobin. The efficiency of the latter, and the activity of metabolism in all tissues, is thus dependent in a great measure upon the presence of thyroid secretion of the blood. Important in this connection are the facts that owing to their high content in phosphorus, all nervous elements, including the nerve-centers, are especially sensitized by thyroid extract, and that their functional activity is correspondingly enhanced. The adrenal centers being subject to this action, as well as all other centers, the thyroid secretion activates metabolism in two ways: (1) By increasing the inflammability of all cells, and (2) by exciting the governing center of general metabolism, that of the adrenals.

The wonderful action of thyroid extract in cretinism now becomes self-evident, the stunted stature recalls inactivity of the pituitary (overactivity of which, we have seen, breeds giants) and inadequate secretory activity of the adrenals. Thyroid extract causes the cretin to grow rapidly. This is evidently due to increased oxygenation since the temperature, previously low, now rises to normal and often beyond. There is, indeed, a greater intake of oxygen, a greater output of carbon dioxide, and increased excretion of nitrogen—all proofs of enhanced metabolism. Every function, every structure, including the skin and hair, shows evidences of development and growth. The nervous system outstrips them all, however, owing to its wealth in phosphorus, we have seen; as a result the mental capacity improves, and ultimately a normal average child is evolved from the idiotic dwarf that was.

This brings us back to the main subject of this address, therapeutics; but therapeutics relieved, in respect to the action of adrenal and thyroid preparations, and also, I may add, of iodine and its salts—of the odium of empiricism. We can now saw HOW *adrenalin,* for example, acts. On entering the blood, it increases its oxygenizing power, as if a temporary exacerbation of acidity of the adrenals had done so; hence its fleeting action. Of *desiccated thyroid* we can also say: It adds to the blood constituents of the thyro-parathyroid secretion, which not only increases the inflammability of the cellular phosphorus under the action of the blood's oxygen, but in doing so it increases incidentally the functional activity of the adrenal center, and, therefore, the oxidizing power of the blood. Both conditions of perfect metabolism being met, the whole organism is endowed with new life when the doses are small but too rapidly consumed when the doses are large.

Moreover, this furnishes the clue to the action of *iodine and its salts,* which, although their action, we have seen, is admittedly unknown, are so valuable in practice that, as stated by Manquat, physicians use them when they are at their wits' ends. As is well known, thyroid extractives become inert when the iodine they contain is abstracted; it is at least in part to this haloid, therefore, that its owes its action. Iodine and its salts act much as do thyroid preparations, therefore—a fact confirmed in practice—but less actively, owing to the absence of the other components of the thyroid secretion, which give them, as suggested by Notkin, the character of a ferment.

The practical feature in this connection is that thyroid extract is capable also of increasing the power of the blood to destroy germs and their toxins. Grueber long ago urged that the defensive constituents of the blood were internal secretions; Wright holds a similar view concerning the opsonins; but neither of these distinguished investigators discovered their source. Now, my own researches have shown that *opsonin* gives the reactions of the thyro-parathyroid secretion; that the oxygenized adrenal secretion, that is to say, the albuminous constituent of hemoglobin, is Ehrlich's *amboceptor,* and that the main constituent of his complement is *trypsin,* the identical ferment through which, according to Metchnikoff, phagocytes kill and digest bacteria.

The process through which a thyroid preparation increases the auto-protective activity of the blood now suggests itself: it increases directly the opsonin which sensitizes what bacteria, toxins, toxic wastes, etc., may be present. By enhancing simultaneously the metabolic activity of all tissues, including the adrenal center, the pancreas and the phagocyte-producing organs, it thus endows the blood, the lymph, and the lymph glands with an excess of their bacteriolytic and antitoxic substances and cells, insures their destruction, and, at the same time, their conversion into eliminable end-products.

This introduces a feature of therapeutics which is at present absorbing the attention of the whole scientific world. How do Koch's tuberculin, Wright's vaccines, Coley's toxins, etc., produce their beneficial effects? Wright stated four years ago, referring to the protective agents of the blood that, if pathologists "knew the laws by which such substances were produced," they could "call forth a production of those substances."

I suggested several years ago that the anterior lobe of the pituitary body was not, as now believed, a secreting structure, but a sensitive organ, which perceived, as it were, any poisonous substance that happened in the blood. I traced this organ throughout the entire animal scale down to such low forms as mollusks. Zoologists having concluded that its purpose was to test the water ingested by these lowly animals for noxious substances, they gave it the suggestive name of *test-organ* or *osphradium.* My opinion that this organ exists in the higher animals was confirmed in Europe recently, Gentès having found histologically in the pituitary, a sensory structure similar to that of the olfactory membrane, a fact which suggests clearly its purpose: To detect the presence in the blood circulating over it, of any poison that it may contain, just as the organ of smell enables us to detect malodorous emanations which compromise the purity of the air.

The manner in which tuberculin and other bacterial poisons produce their effects will now oppear. On entering the blood they circulate throughout the entire body, including the pituitary body and its test-organ. Now, this structure proved histologically to be the sensitive surface—in the sense that the nasal olfactory membrane is a sensitive surface—of the adrenal center, or, better, the adreno-thyroid center, from which arise both the nerve-path, which I have traced to the adrenals, and the nerves which Cyon traced to the thyroid. On the other hand, many investigators have found that during the course of infectious diseases—due, therefore, to bacterial toxins—the thyroid and the adrenals presented the characteristic signs of excessive functional activity, while the fact that these organs serve to destroy blood poisons has been known ever since almost their functions have been studied.

On the whole, evidence from various directions points to the sensitive test-organ and its adreno-thyroid center as the structure excited by tuberculin, Wright's vaccines, Coley's toxins, etc., and to the adrenals and thyroid, which the adreno-thyroid center excites, as the organs which supply the blood with bacteriolytic and antitoxic substances, to which I will refer, henceforth, as "auto-antitoxin," its composition being, as I urged several years ago, the same as that of antitoxin.

The strength of this interpretation is sustained by the fact that the reaction produced by tuberculin and other bacterial vaccines corresponds with that caused by excessive functional activity of the adrenals and thyroid. No one, for example, denies that excessive oxidation underlies the production of fever, and no one will deny that a fever, up to a certain limit, is a protective process; but "even if we grant," as Lazarus Barlow wrote recently, "that fever is beneficial, we are completely ignorant of the manner in which it acts." One can hardly realize that so commonplace a symptom should not as yet, with the enormous aggregate of scientific facts available, have been explained. The cause of this now suggests itself: Neither fever nor any other phase of the immunizing process could be elucidated before the adrenal system, that is to say, the pituitary body, the adrenals, and the thyroid, had been found to fulfill the functions I have described, particularly oxidation.

Essential in this connection is the fact that tuberculin and other bacterial vaccines are used as *remedies*. Interpreted from my standpoint, bacterial vaccines are beneficial, in a given disease, when they increase the efficiency of the body defenses in the manner described. But an important question imposes itself at this point, one bearing particularly upon the branch which interests us all, pharmacology. Can we, with our drugs, also enhance the efficiency of the defensive functions? Let me recall the fundamental principle I have previously urged, that *immunizing medication is the foundation of rational therapeutics.*

And this principle has borne fruit. A letter from an extremely competent and conscientious Virginia physician, Dr. C. F. Brower, received as these lines were being written, may be used to exemplify the tenor of others received: "My results are astonishing to me; and, to my assistant" (who was not as yet familiar with the newer conceptions), "they are astounding and incomprehensible." This refers to results obtained with the most familiar remedies of our pharmacopeia—to a therapeutics in which we can govern the efficiency of our defensive mechanism, just as an engineer can regulate the speed of a locomotive through the throttle valve—a therapeutics, in other words, in which our intelligence, and not empiricism, holds sway.

This does not mean, of course, that our resources are limited to fighting bacteria and the effects of their toxins, toxic wastes, and other poisons. They include the use of measures to counteract insomnia, pain, and other distressing symptoms, and also morbid developments in the course of disease such as cerebral or pulmonary congestion, paralyses of function, etc. To make this clear, I will close my remarks with a brief outline of the two or three main effects through which a few groups of drugs, tonics, analgesics, hypnotics, etc., counteract these morbid phenomena, referring you to the work previously mentioned for the action of other groups, a review of which would too greatly prolong this address.

A few explanatory facts are needed to facilitate your understanding of the principles I am about to submit: 1. The adreno-thyroid center, we have seen, receives the impulses it transmits to the adrenals and thyroids from the sensory test-organ, which resents, as it were, the presence of a poison in the blood, and awakens the protective reaction. To simplify the explanation of the mode of action of the drugs which evokes this defensive process in the blood, I will only mention the adreno-thyroid center, taking it for granted you will understand that it is the sensitive surface of this center, the test-organ, which is primarily excited by the drug. 2. Not all drugs stimulate this organ; some depress it or anesthetize it more or less actively, as will be shown presently. 3. Notwithstanding the fact that most drugs, in therapeutic doses, act primarily upon one or more centers, each drug acts specifically; that is to say, in a manner peculiar to that blood alone, or to the group of drugs to which it belongs.

In the case of one group of agents, those which provoke, through their action upon the adrenal system, the appearance in the blood of an excess of auto-antitoxin, a new term became necessary. That of "antitoxigen" appears to me to convey the intended meaning.

*Antitoxigens.*—The most active antitoxigens in infections and intoxications include some of the drugs now known as alteratives, the physiological action of which, we have seen, has remained obscure. *Thyroid* preparations are very active in this connection since they provide the blood directly, as previously explained, with an excess of opsonin to sensitize the bacterial toxins and other poisons. At the same time, by exciting the adrenal center, they incite the production of the other constituents of auto-antitoxin, and the genesis of phagocytic leucocytes. *Iodine* and the iodides act in the same way, but they provide less opsonin, since they only supply the thyroid and parathyroids with the main agent which enables these organs to increase their production of this sensitizing body. They are, therefore, far less active, in this particular, though they compensate for it, in a measure, by exciting more actively the adreno-thyroid center, thus enhancing oxidation to a greater extent. *Mercury* is well known in this connection, as the widespread use of calomel attests. Its salts act by exciting powerfully the adreno-thyroid center, thus providing the blood more evenly, as it were, than the two preceding agents, with all its defensive constituents. Their well-known action on the liver (since this is the organ in which the antitoxic process is most active) and in infections, especially syphillis, is due to this property.

A remarkable agent in this connection is *creosote,* so effective in pulmonary tuberculosis and pneumonia when judiciously used. Not only does it promote the formation of auto-antitoxin and phagocytes by exciting the adreno-thyroid center, but it depresses the center which regulates the caliber of the arterioles, causing these small vessels to dilate. As a result an excess of blood is admitted into all capillaries, including those of the diseased area, and as this blood is unusually rich in auto-antitoxin and phagocytes, the pathogenic bacteria and their toxins are vigorously attacked.

The solvent property attributed to alteratives is readily explained by the action of the opsonin or thyroid secretion which they directly or indirectly add to the blood, the increased inflammability of the phosphorus contained in morbid products, gummata, for instance, rendering them more amenable to the digestive action of the auto-antitoxin. This property, when exaggerated, becomes a source of danger, when too large doses of either of the above remedies are given, since the red corpuscles and the tissues themselves may then undergo dissolution—observations borne out by the familiar terms "hemolysis" and "autolysis." Again, fever, we have seen, is due to a more or less marked increase of the auto-antitoxin in the blood, but if it exceeds 105 degrees F. to any great extent, the bacteria are not only dissolved, but the red corpuscles are also exposed to dissolution.

*Tonics.*—The most active of these remedies also excite the adreno-thyroid center, but only sufficiently to enhance general nutrition. Practically all these agents, however, influence another center at the same time, usually the vaso-motor center. *Strychnin* is one of these, though it does not excite the adreno-thyroid center as actively as do mercurials, by stimulating also the vaso-motor center, it causes general vaso-constriction, and by thus driving the enriched blood into the tissue capillaries increases general nutrition. The beneficial action of *digitalis* in heart disorders is due to a marked stimulating action on the adreno-thyroid center. It also excites, however, the sympathetic center (to be described presently), which not only governs the caliber of the arterioles, but causes these vessels to propel the blood into the capillaries, and to increase thereby the nutrition of all tissues, including the heart-muscle. Its powerful action on the adreno-thyroid center causes the adrenal secretion to be produced in considerable quantity when large doses are given; as this secretion serves also, as I urged several years ago, to aid the contractions of the right ventricle, an excess of it causes the latter to contract with greater vigor than the left, the pulse becoming dicrotic, as noted by various authors. *Strophanthus, apocynum, convallaria,* and other heart tonics act in a similar way, but with less vigor.

*Quinin,* on the other hand, acts indirectly. It does not excite the adreno-thyroid center, but the vaso-motor center. In large doses it causes such violent general vaso-constriction that the blood is forced into the peripheral capillaries, the brain, the ears, the conjunctiva, etc. Hence the headache, the tinnitus, etc. It protects against malarial infection by driving an excess of blood (including what auto-antitoxin it happens to contain) into the skin, thus augmenting the resistance to infection by the mosquito. Its curative power is due, as is well known, to its direct toxic action upon the plasmodium.

*Hypnotics*—The most active of these agents, that is to say, the true hypnotics, produce their action by markedly depressing the adreno-thyroid center. This is the action of *chloral hydrate*, for instance. As metabolism is thus slowed in all tissues, including the muscular elements of the arteries and veins, these vessels dilate and the blood recedes into the larger trunks, especially those of the splanchnic area. The volume of blood in the brain being reduced, sleep follows. *Paraldehyde, sulphonal,* and *trional* act in the same way, the two last named, however, with greater violence, as shown by the cyanosis they sometimes produce—a self-evident sign of deficient oxygenation, the function over which the adrenals preside. The *bromides* are less efficient as hypnotics but this is because they do not depress the adreno-thyroid center when given in anything except large doses. They depress the vaso-motor center and cause sleep, as does chloral, by causing general vasodilation and recession of blood from the brain.

*Analgesics.*—The cause of the prevailing obscurity as to the manner in which these agents relieve pain, is explained by the fact that the functions of the nerves through which they do so has also remained obscure. Although the vaso-motor center is located in the bulb, electrical stimulation of the pituitary body, as shown by Cyon, Masay, and others, causes an immediate rise of the blood pressure. I found that this was due to the presence in that organ of a center, the search for which had been abandoned many years: that of the sympathetic system; and, moreover, that its purpose was to govern the propelling power of the arterioles which admit arterial blood into all capillaries. With this fact before us the action of analgesics becomes self-evident. *Antipyrin,* for example, relieves pain because it excites the sympathetic center in the pituitary and thereby causes excessive constriction of the arterioles; the volume of arterial blood circulating in the sensory terminals of the diseased area being reduced, the pain ceases. That this mechanism is the true one is shown by Sawadowski's observation that a section across the basal structures, that is to say, between the pituitary and the bulb, causes antipyrin to lose its action. *Acetanilid* and other coal-tar products act in the same way. So do *opium, morphin,* and other opiates. The hypnotic action of large doses of the latter agents is accounted for by the same process, the cerebral arterioles being also constricted and less blood circulating in the brain, sleep is produced.

*Anesthetics.*—These agents differ totally from either hypnotics or analgesics in the manner they produce their effects. Chloroform, by exciting violently the vaso-motor center, first causes general vaso-constriction, and by thus driving an excess of blood forcibly into the capillaries of the cerebro-spinal system, produces the first stage of anesthesia, that of excitement. True anesthesia occurs when the vaso-constriction of both arteries and veins becomes sufficient to produce stasis of the blood in the cerebro-spinal capillaries, and to cause it to become prematurely venous: the functions of the brain being in abeyance, sleep and anesthesia follow. The action of *ether* differs from that of chloroform only in that it does not excite the vaso-motor center as violently. *Nitrous oxide* produces anesthesia by preventing the access of air to the venous blood and the adrenal secretion it contains while passing the air cells: the adrenal secretion having failed to become oxygenized by the alveolar air, does so at the expense of the circulating blood after passing the alveoli. Hence the immediate cyanosis and the rapidity of the anesthesia, the entire cerebro-spinal system being supplied with blood deficient in oxygen.

*Vaso-Cardiac Depressants.*—*Veratrum viride,* the most important of these agents, produces its effects by depressing markedly the vaso-motor center. All the vessels of the body being dilated, the blood recedes from the periphery to the deeper and larger vessels, excessive arterial tension and the resulting resistance of the blood-column to the heart's contractions are counteracted. This is the only remedy whose physiological action has, in my opinion, been known. *Aconite* produces its effect by depressing the sympathetic center, thus causing dilation of the arterioles and the penetration of an excess of blood into the capillary system. When large doses are given this capillary hyperemia excites the peripheral end-organs of sensibility and tingling is caused, besides flushing, headache, etc.

These few examples must suffice to sustain the impression I hope to convey, that if, as stated by Professor Sollmann, therapeutics is now not even an art, but "a confusion," the painstaking labors of a host of investigators have afforded us all the factors necessary to at least elaborate a foundation for a system in which simplicity and precision supplant confusion. Indeed, I must emphasize the fact that although the action of each drug, as I interpret it, is devoid of complexity, it is based on a vast array of evidence—all of which converges towards the solution I urge. The action of mercury, for instance, is sustained by the recorded results of 77 experiments—to say nothing of the clinical data adduced.

Permit me to add that notwithstanding this simplified pharmacology, it is possible to select accurately the remedy that will meet the determining cause of a disease in its own field and more than ever before, counteract its morbid effects. What may we not hope when, with your aid, our knowledge, from the mere rudiments I have to offer will have reached a high degree of perfection? Not only will therapeutics have attained its merited position as a science, but it will have raised medicine itself to so high a plane in the public estimate that no one will even think of trusting health and life to any man or woman other than a scientifically trained physician.

